# Frequency of hyponatremia caused by sodium picosulfate solution when used as a bowel cleansing agent for colonoscopy

**DOI:** 10.12669/pjms.36.7.2376

**Published:** 2020

**Authors:** Tazeen Rasheed, Haris Alvi, Majid Ahmed Shaikh, Faiza Sadaqat Ali, Bader Faiyaz Zuberi, Wara Subhan

**Affiliations:** 1 Dr. Tazeen Rasheed, Assistant Professor, Dow University of Health Sciences, Karachi, Pakistan; 2 Prof. Haris Alvi, MBBS, FCPS. Dow University of Health Sciences, Karachi, Pakistan; 3 Dr. Majid Ahmed Shaikh, Dow University of Health Sciences, Karachi, Pakistan; 4 Dr. Faiza Sadaqat Ali, Senior Registrar, Dow University of Health Sciences, Karachi, Pakistan; 5 Prof. Bader Faiyaz Zuberi, Dow University of Health Sciences, Karachi, Pakistan; 6 Dr. Wara Subhan, Postgraduate trainee, Dow University of Health Sciences, Karachi, Pakistan

**Keywords:** Colonoscopy, Bowel preparation, Sodium Picosulfate

## Abstract

**Objective:**

To determine the frequency of hyponatremia in patients taking Sodium Picosulfate Solution (SPS) solution for bowel preparation prior to colonoscopy and to compare serum sodium levels before and after SPS.

**Methods:**

This interventional study was conducted at Dr. Ruth K. M. Pfau, Civil Hospital Karachi between June 2019 to November 2019. Patients undergoing colonoscopy were included in the study. All patients were given SPS. Two samples of blood for electrolytes were taken, one 30 minutes before taking SPS solution and another 30 minutes before colonoscopy. Paired sample *t-test* was used to determine the difference between serum sodium level before taking the colonoscopy solution and serum sodium level before colonoscopy.

**Results:**

Fifty- four patients fulfilling inclusion criteria were included. Out of the 54 patients 28 (51.9%) were males and 26 (48.1%) were females. Mean sodium levels before taking colonoscopy solution was 139.7 ±3.5 mEq/L and mean sodium level before colonoscopy was 138.9 ±3.8 mEq/L. The difference between serum sodium level before taking SPS colonoscopy solution and before colonoscopy was found to be statistically insignificant (t (53) = 1.308; p = 0.196).

**Conclusion:**

No serious adverse effects were reported in any of our patients. There was no significant difference in the serum sodium level of patients undergoing colonoscopy before taking SPS bowel preparation solution and serum sodium level before colonoscopy.

## INTRODUCTION

Colonoscopy is a useful tool used in modern medicine and it is increasingly being availed for both diagnostic and therapeutic purposes. However, its effectiveness is highly dependent on the quality of bowel cleansing.^1^ The diagnostic effectiveness of colonoscopy depends upon the quality of the preparation.^2^ Optimal bowel preparation leads to shortened caecal intubation time, increased rate of polyp detection with a subsequent increased adenoma detection rate whereas a suboptimal bowel preparation can result in missed small or flat lesions, significant impediment in progression of colonoscope, reduced caecal intubation rates and more sedatives and analgesics being required.^3^ The most frequently used solutions for colonoscopy preparation are polyethylene glycol (PEG) Sodium Phosphate and Sodium Picosulfate (SPS).^4^ Bowel preparation for colonoscopy can lead to electrolyte abnormalities such as hyponatremia which may cause serious neurological consequences such as seizures, loss of consciousness and eventually coma.^5^ SPS acts in the colon as a stimulant laxative and increases the frequency and strength of peristalsis thereby causing diarrhoea.^6^ However, there are some cases of hyponatremia reported due to its use. Fluid and electrolyte disturbances can occur more commonly in patients with risk factors, such as old age, use of SSRIs and thiazide diuretics, chronic kidney disease, congestive heart failure or with a history of electrolyte abnormalities.^1^

There are several case reports on hyponatremia after colonoscopy preparation solution, but none are reported from Pakistan. Our objective was to determine frequency of hyponatremia as a result of bowel preparation that will create awareness among colonoscopists regarding this electrolyte imbalance. Thus, it will increase patient safety and better management in patients undergoing this procedure.

## METHODS

This interventional study was conducted at Dr. Ruth K. M. Pfau., Civil Hospital Karachi between June 2019 to November 2019. Patients satisfying inclusion/ exclusion criteria were included after written informed consent. Approval (IRB-1245/DUHS/Approval/2019/47, Dated: June 1^st^, 2019) was taken from Institutional Review Board of Dow University of Health Sciences.

Sample size was calculated using PASS 11 software using a Two-sided Z- test S(P0) using a difference (PI-P0) of 0.24. The power of analysis is 95 % and p-value 0.05. The sample size was calculated to be 42. Considering a 20% dropout rate the dropout inflated enrollment sample size was 53. Sampling technique was non-probability consecutive sampling.

Patients of both genders of age between 16-60 years undergoing colonoscopy were included. Patients with Chronic kidney disease, patients taking diuretics for any reason, patients who failed to follow the preparation protocol and patients of hypothyroidism were excluded.

All patients were given two doses of SPS of 45ml each diluted in 400 ml of water at 12 PM on the day preceding colonoscopy. They were advised to drink two more glasses of 400 ml of water in the next hour. Second dose of SPS of same strength and in similar dilution, was given at 6:00 PM the same day, followed by two glasses of water. They were advised to take liquid diet for 24 hours before colonoscopy and nil per oral orders for 4 hours before colonoscopy. Two samples of blood for electrolytes were taken, one 30 minutes before taking SPS solution and another 30 minutes before colonoscopy. Hyponatremia was defined as serum sodium level <135 mEq/L^7^ and Hypernatremia was defined as serum sodium levels >145 mEq/L.^8^

### Data Analysis Procedure

Data was analyzed using SPSS version 25. Mean ±SD were reported for age and sodium levels. Mean age was compared with gender using student’s t-test. Paired sample t-test was used to determine the difference between serum sodium level before taking the colonoscopy solution and serum sodium level before colonoscopy. Values of serum Na+ before and after were recoded into new variables using the cutoff criteria of < 135 mEq/L labeling them having hyponatremia or not. Frequency of hyponatremia before and after SPS was determined and compared using χ^2^ test. Change in Na+ levels were determined by calculating the difference between Na+ levels before and after SPS (negative values showed decrease and positive values showed increase in Na+ levels after SPS). P-value of ≤ .05 was taken as significant.

## RESULTS

In this study fifty- four patients undergoing colonoscopy and fulfilling inclusion criteria were included. Mean ±SD of age of the patients was 40.1 ±15.2 years. Out of the 54 patients 28 (51.9%) were males and 26 (48.1%) were females. Mean age of males was 38.6 ±15.3 years while that of females was 41.8 ±15.3 years. The difference in age among genders was not statistically significant (p = 0.452; df 52; 95% CI -11.5 to 5.2).

Mean sodium levels before taking colonoscopy solution was 139.7 ±3.5 mEq/L. Out of these 5 (9.3%) had levels <135 mEq/L, i.e., they were having hyponatremia before taking SPS while one (1.9%) was having hypernatremia before SPS. Mean sodium level after SPS was 138.9 ±3.8 mEq/L. Hyponatremia was seen in 6 (11.1%) patients while hypernatremia was seen in 2 (3.7%) patients after SPS. The difference between mean serum sodium levels before and after SPS was found to be statistically not significant [t (53) = 1.308; p = 0.196]. No change in Na+ levels was observed in 5 (9.3%) after SPS solution, Na+ levels increased in 15 (27.8%) from index value and decreased in 34 (63%) patients from index value after SPS. Details are given in Table-I.

**Table-I:** Paired Sample Statistics of Serum Sodium before and After Taking Colonoscopy Solution.

	Mean	SD*	SEM†	95% CI*‡*	P Value
Na+ Before Solution	139.70	3.450	.469	-0.415 to 1.970	0.196
Na+ After Solution	138.93	3.828	.521		

Standard Deviation, ^†^Standard Error of Mean, ^‡^Confidence Interval

## DISCUSSION

Colonoscopy is widely performed nowadays for both diagnostic and therapeutic purposes. It is the gold standard investigation for screening of colorectal cancer and millions of procedures are performed every year throughout the world.^9^ An adequately prepared colon is an important prerequisite for the success of colonoscopy since the quality of bowel cleansing is a crucial factor in determining the speed, difficulty and completeness of colonoscopy.^10^ Colonoscopy solutions are commonly prescribed for bowel preparation. Several colonoscopy solutions are available. Case reports are available showing that bowel preparation solutions for colonoscopy can cause electrolyte abnormalities. The risk of electrolyte imbalances depends on the type of bowel preparation solution used, age of patient and other comorbidities.^11^

SPS colonoscopy preparation solution is commonly used in our setup. SPS is a prodrug that is converted in the colon by bacteria to its active metabolite that is 4,4′-dihydroxydiphenyl-(2-pyridyl) methane. It increases the force and frequency of peristalsis thereby promoting bowel evacuation.^12^ There are case reports that SPS bowel preparation solutions can cause severe hyponatremia in some patients.^11^,^13^ In a study by Cohen CD et al. hyponatremia was assessed in 40 patients before and after colonoscopy in which 7.5% of patients developed hyponatremia after colonoscopy, with a concomitant increase in arginine vasopressin(AVP).^14^ The release of AVP as a result of volume depletion secondary to bowel preparation has been described as a triggering factor for hyponatremia in this clinical setting. Hyponatremia is a serious complication which if occurs rapidly can lead to death due to its associated sequelae.

In our study we aimed to determine the frequency of hyponatremia in patients taking SPS solution for bowel cleansing prior to colonoscopy. In our study there was no significant difference between serum sodium level before and after taking SPS. Our findings were supported by a study by Rahman A et al. in which it was reported that there was no significant change in the mean sodium and glucose levels in patients who were given SPS solution. The results of the same study also reported hypokalemia and hypocalcemia, but these returned to baseline within 24 hours.^15^ However, in another study by Weir MA et al. it was reported that SPS bowel preparation solution was associated with a higher risk of hospitalization with hyponatremia as compared to polyethylene glycol solution.^13^

Hyponatremia has been described as ‘a possible but forgotten consequence of bowel preparation for colonoscopy.^16^ Colonoscopists should be acquainted with this complication because it can result in electrolyte abnormalities which in turn can lead to serious neurological sequelae.

### Limitations of the study

Ours was a single center study with a small sample size. We did not evaluate our patients for electrolyte abnormalities other than sodium due to SPS.

## CONCLUSION

SPS is a safe and easily tolerable bowel preparation solution. No serious adverse effects and no significant changes in serum sodium levels were reported in any of the patient. However, its safety in patients with preexisting electrolyte abnormalities and comorbidities cannot be established. More research is needed to establish its safety in patients with electrolyte abnormalities, in patients taking drugs or suffering from comorbidities that effect serum electrolytes.

### Authors’ Contribution:

**All Authors:** are responsible for data and study integrity.

**TR:** Did coloscopies, Study Conception and is responsible for data integrity.

**HA:** Data collection, initial manuscript writing.

**MAS:** Did colonoscopies, manuscript review and statistical analysis.

**FSA:** Manuscript writing and statistical analysis.

**BFZ:** Did colonoscopies, corrections and final approval of manuscript.

**WS:** Data collection, initial manuscript writing.

## References

[1] Samad N, Fraser I (2017). Severe Symptomatic Hyponatremia Associated with the Use of Polyethylene Glycol-Based Bowel Preparation. Endocrinol Diabetes Metab Case Rep.

[2] Arora MOP (2008). Use of Powder Peg-3350 as a Sole Bowel Preparation:Clinical Case Series of 245 Patients. Gastroenterol Hepatol.

[3] Romero RV MS (2013). Factors Influencing Quality of Bowel Preparation for Colonoscopy. World J Gastrointest Endosc.

[4] Alan Barkun NC, Enns R, Marcon M, Natsheh N (2006). Co Pham, Dan Sadowski, Stephen Vanner Commonly Used Preparations for Colonoscopy. Efficacy, Tolerability and Safety - A Canadian Association of Gastroenterology Position Paper. Can J Gastroenterol.

[5] Baeg MK, Park JM, Ko SH, Min GJ, Lee KJ, Yang JH (2013). Seizures Due to Hyponatremia Following Polyethylene Glycol Preparation;A Report of Two Cases. Endoscopy.

[6] Koshitani T KM, Yoshikawa T (2004). Bowel Preparation for Colonoscopy Using Standard Vs Reduced Doses of Sodium Phosphate:A Single-Blind Randomized Controlled Study. World J Gastrointest Endosc.

[7] Verbalis JG, Goldsmith SR, Greenberg A, Korzelius C, Schrier RW, Sterns RH (2013). Diagnosis, Evaluation and Treatment of Hyponatremia:Expert Panel Recommendations. Am J Med.

[8] Adrogue HJ, Madias NE (2000). Hypernatremia. New Eng J Med.

[9] Seeff LC RT, Shapiro JA, Nadel MR, Manninen DL, Given LS, Dong FB, Winges LD, McKenna MT (2004). How Many Endoscopies Are Performed for Colorectal Cancer Screening?Results from Cdc's Survey of Endoscopic Capacity. Gastroenterol.

[10] Froehlich F WV, Gonvers JJ, Burnand B, Vader JP (2005). Impact of Colonic Cleansing on Quality and Diagnostic Yield of Colonoscopy:The European Panel of Appropriateness of Gastrointestinal Endoscopy European Multicenter Study. Gastrointest Endosc.

[11] Costa JM, Soares JB (2017). Symptomatic Hyponatremia after Bowel Preparation:Report of Two Cases and Literature Review. Acta Med Port.

[12] Hoy SM SL, Wagstaff AJ (2009). Sodium Picosulfate/Magnesium Citrate:A Review of Its Use as a Colorectal Cleanser. Drugs.

[13] Weir MA, Fleet JL, Vinden C, Shariff SZ, Liu K, Song H (2014). Hyponatremia and Sodium Picosulfate Bowel Preparations in Older Adults. Am J Gastroenterol.

[14] Cohen CD KC, Schiemann U, Schroppel B, Siegert S, Rascher W, Gross M (2001). Hyponatraemia as a Complication of Colonoscopy. Lancet.

[15] Rahman A, Hookey LC (2012). Serial Monitoring of the Physiological Effects of the Standard Pico-Salax®Regimen for Colon Cleansing in Healthy Volunteers. Can J Gastroenterol.

[16] Scarpignato C, Blandizzi C (2014). Editorial:Hyponatremia - a Possible but Forgotten Consequence of Bowel Preparation for Colonoscopy. Aliment Pharmacol Ther.

